# ASC Regulates Subcutaneous Adipose Tissue Lipogenesis and Lipolysis via p53/AMPKα Axis

**DOI:** 10.3390/ijms231710042

**Published:** 2022-09-02

**Authors:** Hong Chen, Qilin Pei, Linfen Tao, Jing Xia, Guocai Lu, Ying Zong, Wenhua Xie, Wanqing Li, Chenglong Huang, Ting Zeng, Xinyu Yu, Weixuan Wang, Gaojun Chen, Song Yang, Rui Cheng, Xi Li

**Affiliations:** 1 Institute of Life Sciences, School of Basic Medicine, Chongqing Medical University, Chongqing 400016, China; 2 Department of Laboratory Medicine, School of Medical Technology and Engineering, Fujian Medical University, Fuzhou 350001, China; 3 Department of Health Toxicology, Faculty of Naval Medicine, Second Military Medical University, Shanghai 200433, China

**Keywords:** ASC, lipogenesis, lipolysis, p53, AMPKα

## Abstract

Obesity has become an extensive threat to human health due to associated chronic inflammation and metabolic diseases. Apoptosis-associated speck-like protein (ASC) is a critical link between inflammasome and apoptosis-inducing proteins. In this study, we aimed to clarify the role of ASC in lipid metabolism. With high-fat diet (HFD) and knockout leptin gene mice (*ob*/*ob*), we found that ASC expression in subcutaneous adipose tissue (SAT) correlated with obesity. It could also positively regulate the reprogramming of cellular energy metabolism. Stromal vascular fractions (SVF) cells derived from the SAT of *Asc^−/−^* mice or SVF from wild-type (WT) mice transfected with ASC siRNA were used to further investigate the underlying molecular mechanisms. We found ASC deficiency could lead to lipogenesis and inhibit lipolysis in SAT, aggravating lipid accumulation and impairing metabolic balance. In addition, our results showed that p53 and AMPKα expression were inhibited in SAT when ASC level was low. p53 and AMP-activated protein kinase α (AMPKα) were then assessed to elucidate whether they were downstream of ASC in regulating lipid metabolism. Our results revealed that ASC deficiency could promote lipid accumulation by increasing lipogenesis and decreasing lipolysis through p53/AMPKα axis. Regulation of ASC on lipid metabolism might be a novel therapeutic target for obesity.

## 1. Introduction

Obesity is one of the most significant clinical problems among people of all ages. Obesity strongly correlates with metabolic diseases such as type 2 diabetes mellitus and non-alcoholic fatty liver disease [1,2]. When caloric intake exceeds energy expenditure, the body stores energy in adipocytes as triglycerides (TAGs), which promotes lipid accumulation and causes obesity [3].

Adipose tissue is vital for the regulation of lipid metabolism, and the dynamic balance of lipogenesis and lipolysis in adipose tissue is crucial for maintaining systemic energy homeostasis [4]. The enzymes involved in lipogeneses, such as acetyl CoA carboxylase (ACC) and FA synthase (FASN) [5], are responsible for storing energy in the form of triacylglycerol in adipocytes. Meanwhile, the enzymes that participate in lipolysis, such as hormone-sensitive lipase (HSL) and adipose triglyceride lipase (ATGL) [6] regulate catabolism of triacylglycerol. The dysregulation of the lipogenesis and lipolysis process is closely associated with metabolic disorders and adipose tissue malfunction, leading to insulin resistance and ectopic lipid accumulation.

The dysregulation of the lipogenesis and lipolysis in adipose tissue is always involved in obesity, which is accompanied by chronic inflammation [4]. As an integral part of inflammation, the inflammasome plays a part in the initiation and development of obesity [7]. Apoptosis-associated speck-like protein (ASC) could assemble the inflammasome as a central scaffold protein to activate proinflammatory substrates [8]. In addition, ASC could also interact with apoptosis-inducing p53 and Bcl-2-associated X protein (BAX) to form a pro-apoptotic complex and further enhance apoptosis pathway [9].

As the results of one notable report indicated, in the subcutaneous adipose tissue (SAT) samples isolated from postmenopausal obese women, the ASC expression of adipocytes was increased [10]. Their data showed that ASC expression is associated with obesity. We also observed in mice that the ASC expression in SAT was increased in obesity. Most researchers explained the upregulated ASC was due to the increase of inflammation. However, we noticed that one research group demonstrated that ASC could regulate lipids deposition in bone marrow macrophages (BMMs) [11]. Meanwhile, our previous data found that ASC expression in SAT was upregulated during fasting. Fasting state has been found to decrease inflammasome activation and blunt inflammation [12,13]. Therefore, in fasting state, ASC-mediated function was distinct and independent from the inflammasome. Thus, the correlation between ASC and energy metabolism in adipocytes need further clarification.

In this study, our experiments demonstrated that ASC deficiency could promote lipogenesis and inhibit lipolysis, encouraging lipid accumulation in vivo and in vitro. Moreover, we found that these effects were mediated by p53/AMPKα axis. Our findings implied that ASC is a potential candidate for obesity and metabolic disease treatment.

## 2. Results

### 2.1. ASC Expression Was Correlated with Obesity and Energy Metabolism

**Figure 1:**
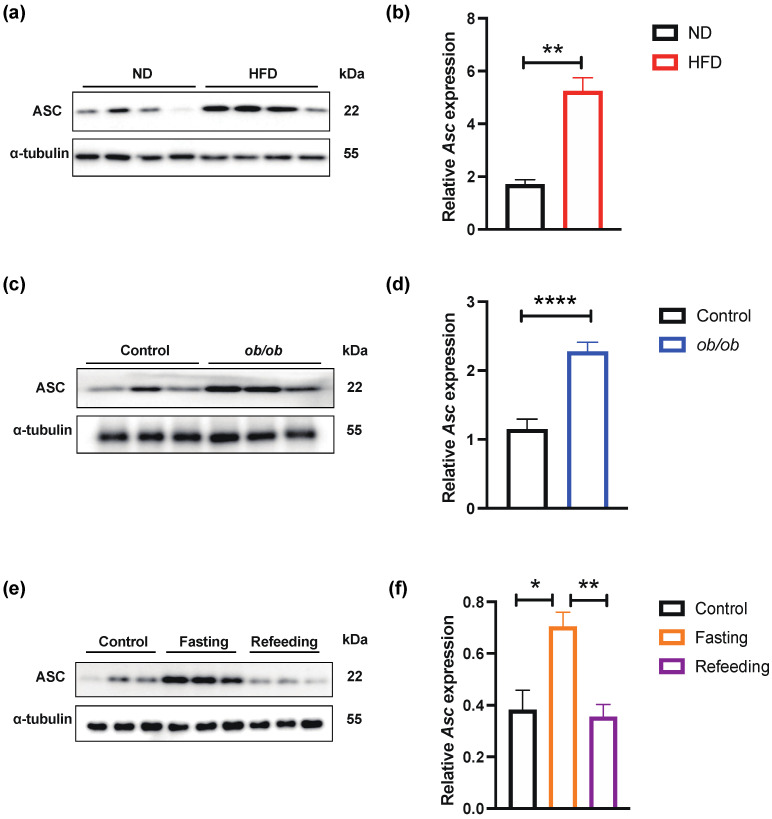
ASC expression in subcutaneous adipose tissue was increased in obese and fasting male mice. (**a**,**b**) Western blot and RT-qPCR analysis of expression of ASC in SAT from ND and HFD mice (n = 4/group) (Black-ND mice, Red-HFD mice); (**c**,**d**) Western blot and RT-qPCR analysis of SAT ASC expression in *ob*/*ob* mice (n = 9/group) (Black-Control mice, Blue-*ob*/*ob* mice); (**e**,**f**) Western blot and RT-qPCR analysis of SAT ASC expression in three groups of WT C57 mice were either not treated (Control), fasted for 24 h (Fasting), or followed by refeeding for 24 h (Refeeding) (n = 6/group) (Black-Control mice, Orange-Fasting mice, Purple-Refeeding mice); * *p* < 0.05, ** *p* < 0.01, **** *p* < 0.0001.

Adipose tissue is the energy metabolism organ for storing TAGs which has a critical regulatory role in obesity and metabolic diseases. We detected ASC expression using obese mice fed with a high-fat diet (HFD) for 21 weeks. The Western blotting (WB) and real-time quantitative (RT-qPCR) showed that the protein expression and mRNA levels of ASC were markedly upregulated in SAT (Figure 1a,b). Consistent with these findings, we also detected the expression of ASC in SAT using knockout leptin gene mice (*ob*/*ob*). The WB and RT-qPCR also showed similar results in the SAT of *ob*/*ob* mice compared with littermate (LM) mice (Figure 1c,d). These findings suggested a correlation between the change of ASC expression level in SAT and the occurrence of obesity. 

ASC may affect the occurrence and development of obesity by influencing the lipid metabolism of adipocytes. To further investigate the function of ASC in adipocytes, the SAT of wild-type (WT) mice were examined under different experimental conditions: not treated, fasted for 24 h, or followed by refeeding for 24 h to observe the expression of ASC. By WB and RT-qPCR validation, we found a significant upregulation of ASC expression in SAT after 24 h of fasting, while at refeeding, ASC expression returned to normal levels (Figure 1e,f). Since fasting could cause adipocytes to undergo reprogramming of cellular energy metabolism, these data suggested that ASC may be directly involved in adipocyte metabolic regulation and lipid metabolism remodeling. These findings indicated that altered ASC expression might play an essential role in the energy metabolism of SAT during the development of obesity. The expression of ASC may be a new therapeutic target for treating obesity-induced insulin resistance and other complications.

### 2.2. ASC Deficiency Regulated Metabolism In Vivo by Promoting Lipogenesis and Inhibiting Lipolysis in SAT

**Figure 2:**
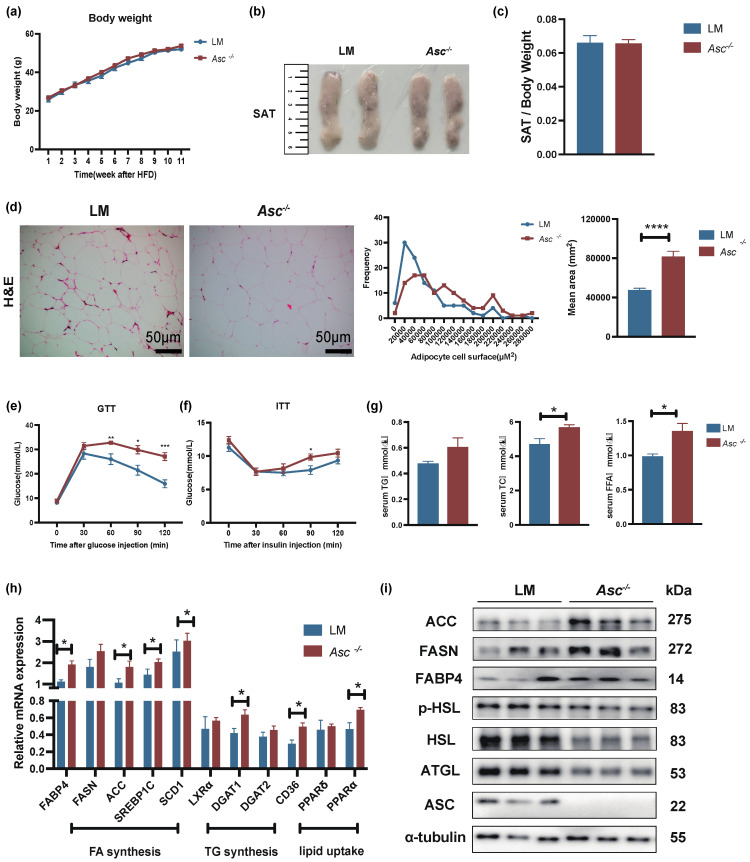
Increased lipogenesis and decreased lipolysis were observed in subcutaneous adipose tissue of *Asc^−/−^* mice fed with HFD. (**a**) Body weight change of LM (n = 8/group) and *Asc* knockout (*Asc^−/−^*) mice (n = 10/group), fed with HFD (12 weeks). (**b**) SAT groups are as described in (**a**). (**c**) Fat index (ratio of SAT weight to whole body weight of indicated mice. (**d**) H&E and quantification of SAT cell size, scale bar 50 µm. (**e**,**f**) GTT and ITT analysis of indicated mice. (**g**) In indicated mice, plasma concentrations of triglyceride (TG), total cholesterol (TC), and free fatty acids (NEFA) at baseline. (**h**) mRNA expression of lipogenesis genes in SAT from indicated mice. (**i**) Western blot analysis of lipogenesis (ACC, FASN, FABP4) and lipolysis (p-HSL, HSL, ATGL) proteins in SAT. All the mice were male and fed with HFD for 12 weeks if not indicated otherwise. (n = 8–10/group) (Red-LM mice, Blue-*Asc^−/−^* mice). * *p* < 0.05, ** *p* < 0.01, *** *p* < 0.001 and **** *p* < 0.0001.

To investigate the influence of ASC on energy metabolism and obesity in vivo, we employed *Asc* gene knockout mice (*Asc^−/−^*) and LM mice born with the same generation as the control. First, we examined the weight change in LM and *Asc^−/−^* mice and found that there was no significant change between LM and *Asc^−/−^* mice fed with HFD for 12 weeks (Figure 2a). Simultaneously, the SAT distribution had no significant difference (Figure 2b,c). Interestingly, the cell size of adipocytes in SAT from *Asc^−/−^* mice was increased compared with LM mice (Figure 2d).

We also assessed glucose homeostasis in these mouse models. The glucose tolerance test (GTT) and insulin tolerance test (ITT) results indicated that ablation of ASC impaired glucose tolerance (Figure 2e) and insulin sensitivity (Figure 2f), resulting in insulin resistance. In addition, not only changes in glucose metabolism but also increased expression of lipid levels in serum. We found ASC deficiency increased basal plasma concentrations of total cholesterol (TC) and free fatty acids (NEFA) (Figure 2g). These data showed that SAT increased lipid accumulation in *Asc^-/-^* mice. Therefore, we wondered whether the absence of ASC affects mouse lipid metabolism. We detected lipogenic genes and proteins in SAT. Surprisingly, we found that the genes coding for lipogenesis were upregulated in SAT of *Asc^−/−^* mice compared with LM (Figure 2h). Meanwhile, the protein levels of FASN, ACC (key transcript factor of lipogenesis) [5], and the fatty acid-binding proteins 4 (FABP4, which is the central role of lipid chaperones) [5,14] were significantly increased in the SAT of *Asc^−/−^* mice compared with LM (Figure 2i). We also found that the protein level of ATGL, phosphor-HSL, and HSL (essential protein of lipolysis) [6,15] were markedly decreased in SAT of *Asc^-/-^* mice (Figure 2i).

### 2.3. Ablation of ASC Promoted Lipogenesis and Suppressed Lipolysis In Vitro

**Figure 3:**
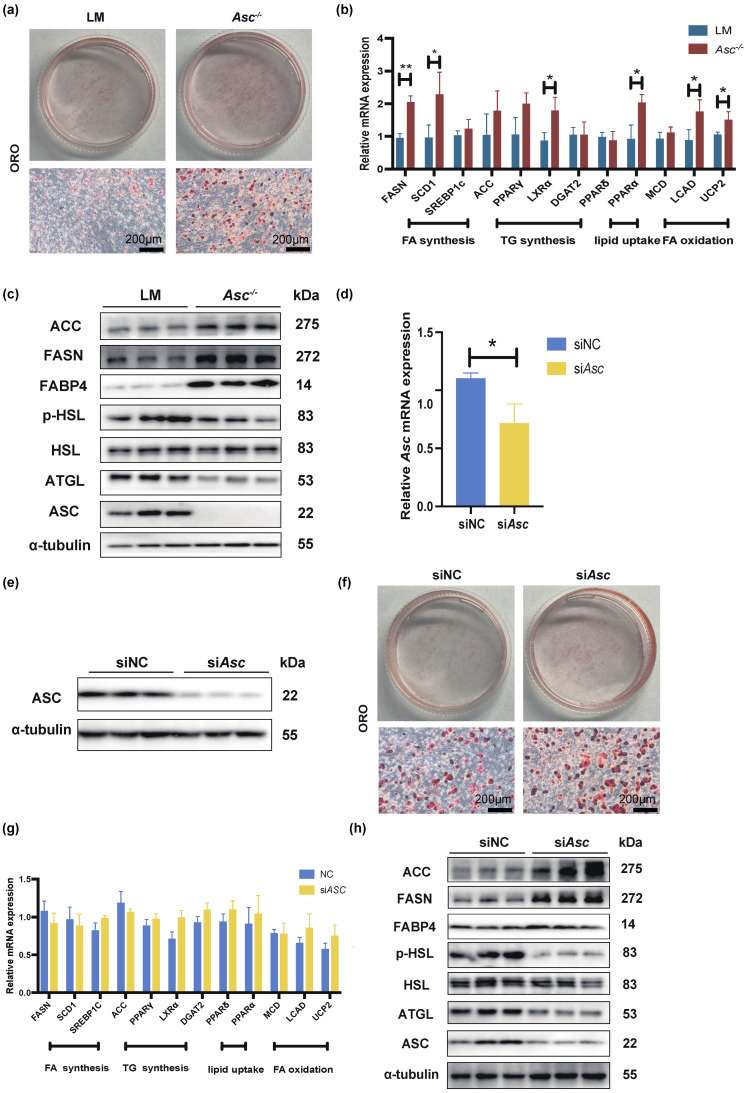
Ablation of ASC in stromal vascular fractions (SVF) cells improved the lipogenesis but impaired lipolysis. (**a**–**d**) Primary SAT SVF cells of LM and *Asc^−/−^* mice (Red-LM mice, Blue-*Asc^−/−^* mice). (**a**) Oil Red O staining of the 6th day under lipogenesis inducement treated primary SAT SVF cells, separated from LM and *Asc^−/−^* mice, scale bar 200 µm. (**b**) The expression of lipogenesis genes was analyzed by RT-qPCR. (**c**) Expression of lipogenesis (ACC, FASN, FABP4) and lipolysis (p-HSL, HSL, ATGL) protein in indicated cells. (**d**–**h**) Primary SAT SVF cells separated from WT mice were transfected with siRNA targeting for ASC on the 4th day (Blue-NC, Yellow-si *Asc*). (**d**,**e**) mRNA and protein level of ASC in indicated cells. (**f**) Oil Red O staining of the 6th day under lipogenesis inducement treated in indicated cells, scale bar 200 µm. (**g**) Expression of lipogenesis genes in indicated cells. (**h**) Expression of lipogenesis (ACC, FASN, FABP4) and lipolysis (p-HSL, HSL, ATGL) protein in indicated cells. Data are presented as means ± SD from three independent experiments. * *p* < 0.05 and ** *p* < 0.01.

The above data showed that ASC deficiency could cause lipid metabolic disorders, so we further explored whether the ablation of ASC also leads to such results in vitro. Previous data found that ASC deletion affects lipid metabolism, mainly in adipocytes, and inflammasomes are mainly expressed in macrophages; therefore, we developed pre-adipocytes derived from stromal vascular fractions (SVF) cells of LM and *Asc^−/−^* mice. The induction of adipocyte culture was tested through a standard protocol that employs a cocktail of insulin, dexamethasone, and 3-isobutyl-1-methylxanthine. SVF cells displayed substantial lipid accumulation 6d after induction of differentiation. Oil Red O staining showed that lipid content of *Asc^−/−^* mice SVF was increased compared with LM (Figure 3a). We also determined lipogenesis mRNA and protein level; the results of RT-qPCR and WB showed that lipogenesis mRNA and protein expression increased in *Asc^−/−^* mice in SVF cells (Figure 3b,c). The phosphor-HSL, HSL, and ATGL protein level of *Asc^−/−^* mice in SVF cells were lessening than LM (Figure 3c).

We evaluated whether ASC expression directly modulated lipogenesis and lipolysis gene expression in mature adipocytes and excluded the effect of adipogenesis in *ASC* knockout mice. Thus, we transfected ASC siRNA into WT mice primary SVF cells that were ready to differentiate into mature adipocytes, thereby observing whether the ablation of ASC affected on the lipid metabolism of mature adipocytes. As RT-qPCR and Western blot showed, ASC mRNA and protein levels were downregulated in SAT SVF cells by ASC siRNA (Figure 3d,e), and we found the same expression trend of lipogenic genes of WT SVF cells with ASC siRNA (Figure 3f–h).

### 2.4. Ablation of ASC Promoted Lipogenesis and Inhibited Lipolysis by Regulating p53/AMPKα Axis in SAT

**Figure 4:**
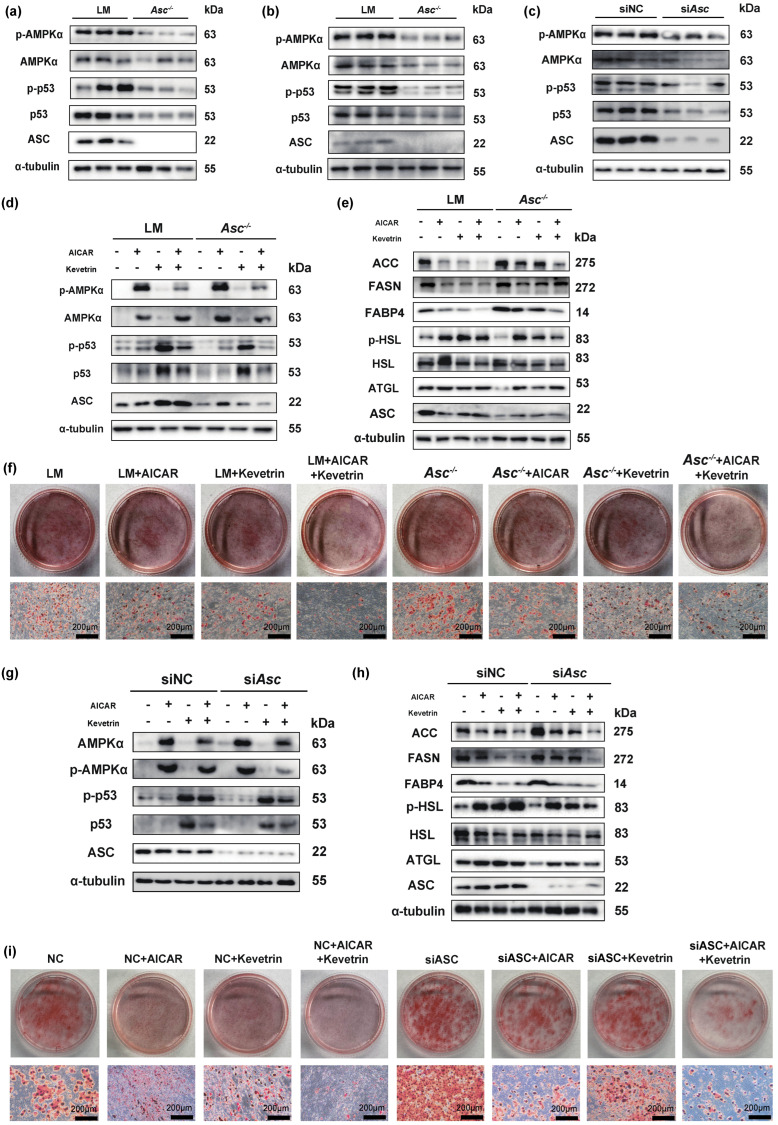
ASC defection promoted lipid accumulation through p53/AMPKα axis. (**a**) Protein expression of phosphor-p53, p53, phosphor-AMPKα, and AMPKα in SAT of LM and *Asc^−/−^* mice fed with HFD (12 weeks). (**b**) Protein expression of phosphor-p53, p53, phosphor-AMPKα, and AMPKα in primary SVF cells separated from SAT of LM and *Asc^−/−^* mice (n = 3 per group). (**c**) Protein expression of phosphor-p53, p53, phosphor-AMPKα, and AMPKα in primary SVF cells separated from WT mice SAT transfected with ASC siRNA (n = 3 per group). (**d**) Protein expression of phosphor-p53, p53, phosphor-AMPKα, and AMPKα in primary SVF cells separated from LM and *Asc^−/−^* mice SAT, with AICAR or Kevetrin treatment (n = 3 per group). (**e**) Protein expression of lipogenesis (ACC, FASN, FABP4) and lipolysis (p-HSL, HSL, ATGL) in primary SVF cells separated from LM and *Asc^−/−^* mice SAT, with AICAR or Kevetrin treated (n = 3 per group). (**f**) Oil Red O staining of the 6th day under lipogenesis inducement treated in primary SVF cells, separated from LM and *Asc^−/−-^* mice, with AICAR or Kevetrin, treated (n = 3 per group), scale bar 200 µm. (**g**) Protein expression of phosphor-p53, p53, phosphor-AMPKα, and AMPKα in primary SVF cells separated from WT mice, with ASC siRNA, AICAR, or Kevetrin treatment (n = 3 per group). (**h**) Protein expression of lipogenesis (ACC, FASN, FABP4) and lipolysis (p-HSL, HSL, ATGL) in primary SVF cells separated from WT mice, with ASC siRNA, AICAR, or Kevetrin treated (n = 3 per group). (**i**) Oil Red O staining on the 6th day under lipogenesis inducement in primary SVF cells separated from WT mice, with ASC siRNA, AICAR, or Kevetrin treatment (n = 3 per group). (AICAR was used at 500 µM for 5 h, Kevetrin was used at 340 µM for 24 h), and the scale bar was 200 µm. Data are presented as means ± SD from three independent experiments.

To explore the mechanism of ASC deficiency promoting lipid accumulation, we detected the p53 and phosphor-p53 expression which is the downstream molecule of ASC [16,17]. We found p53 and phosphor-p53 decreased in SAT of *Asc^−/−^* mice fed with HFD (Figure 4a). AMP-activated protein kinase α (AMPKα), a critical sensor to maintain energy balance and regulate lipogenesis and lipolysis [18,19], could be regulated by p53 [20]. Therefore, we speculated that ASC deletion might further inhibit the level of AMPKα by downregulating the expression of phosphor-p53 level. We found the protein level of phosphor-AMPKα and AMPKα decreased in SAT of *Asc^−/−^* mice fed with HFD, consistent with the hypothesis (Figure 4a). To further validate whether this hypothesis holds in in vitro experiments, we (1) analyzed the SVF cells of *Asc^−/−^* mice and (2) used SVF cells of WT mice which were treated with ASC siRNA to verify the expression of phosphor-p53, p53, phosphor-AMPKα, and AMPKα after ASC deletion using two different knockdown methods, consistent with previous speculations (Figure 4b,c).

To further determine whether ASC regulates lipogenesis and lipolysis through p53/AMPKα axis, p53/AMPKα activators were applied. AMP analogue activator of AMPK (AICAR, Selleck, Houston, TX, USA, 500 µM) [21], activator of p53 (Kevetrin, Selleck, Houston, TX, USA, 340 µM) [22], were added 5 h before cell harvest to counteract the effects of ASC on AMPKα and p53 expression in SVF cells. We observed that the addition of AICAR and Kevetrin upregulated phosphor-AMPKα, AMPKα, phosphor-p53, and p53 protein levels (Figure 4d,g). As expected, AICAR and Kevetrin partially weakened lipogenesis protein expression and increased the lipolysis protein levels (Figure 4e,h); namely, they inhibited lipid accumulation (Figure 4f,i). These results suggested that the effects of ASC deficiency on lipogenic proteins are partially reversed under conditions in which AMPKα and p53 are activated. 

## 3. Discussion

ASC was described as a scaffold protein to form an aggregate during apoptosis cell death [23] or form inflammasome. Recent evidence unveiled that ASC function is not only related to inflammation but also involved in energy metabolism in mice [24,25,26]. Meanwhile, in the 3T3-L1 cell line, inflammatory cytokines could induce NACHT, LRR, and PYD domains-containing protein3 (NLRP3) and caspase-1 expression without affecting ASC expression [10,27]. This also suggested the function of ASC in adipocytes was independent of the inflammasome. In the present study, we found that ASC deficiency promotes lipogenesis, inhibits lipolysis, and increases lipid accumulation via p53/AMPKα in SAT.

It has been shown that *Asc^−/−^* mice fed with HFD lost body weight, and their serum insulin levels were reduced, thereby promoting insulin sensitivity [24,25]. However, we found no significant change in body weight in *Asc^−/−^* mice comparing with littermates, but ASC deficiency exacerbated glucose intolerance with HFD. These findings may seem paradoxical, which may be due to the different feeding conditions and the difference between the control mice. In Stienstra’s study, the mice were fed with 45% kcal HFD for 16 weeks, while in our experiments, *Asc^−/−^* mice were fed with 60% kcal HFD for 12 weeks. Although the HFD we used was purchased from the same company, the difference in composition between 45% kcal and 60% kcal HFD, including the percentages of fat, carbohydrates, proteins, and sucrose, can affect mice’s metabolism. Given the complex metabolic microenvironment in adipose tissue, differences in HFD content and feeding time could result in different phenotypes. Moreover, Stienstra et al. used wild-type mice as control. However, the ideal control is to use littermates as we did in the experiments to ensure as much as possible that their genetic background, growth environment, etc. are the same. These experimental differences may be why our findings are inconsistent with previous studies.

Additional evidence suggested that the absence of ASC did not prevent obesity-induced macrophage infiltration into adipose tissue. This research also pointed out that ASC may mediate adipocyte hypertrophy and regulate macrophage infiltration independently of inflammasome [25]. However, in our study, flow cytometry (FCM) analysis showed that in the SAT of *Asc^−/−^* mice fed with HFD, the proportion of M1 adipose tissue macrophages (ATMs) decreased, and the proportion of M2 ATMs increased, resulting in a significant decrease in M1/M2 ratio (Figure S1a,b). Furthermore, we found the proinflammatory cytokines interleukin-1β (IL-1β) and the phosphorylation of Nuclear factor- kappa B (NF-κB, p65, the critical regulator of inflammatory) was also inhibited in the SAT of *Asc^−/−^* mice fed with HFD as expected (Figure S1c). 

It has been shown that ASC regulates lipid deposition in macrophages and results in atherosclerosis [11]. Some research reported that the lipolysis process was induced acutely in fasting by β-adrenergic signaling and inhibited in feeding by insulin [28,29]. In adipocytes, lipogenesis was activated by high carbohydrate supply and the actions of insulin. Our work demonstrated that the expression of ASC was positively correlated with lipolysis and negatively correlated with lipogenesis in the fast and refeed state. These data suggested that ASC may be involved in regulating lipid metabolism in different physiological (fasting-refeeding) and pathological (high-fat diet-induced obesity) conditions.

AMPKα, a core sensor of cellular energy status that regulates lipid metabolism by regulating FASN, ACC, HSL, and ATGL expression [30,31], could be activated by phosphor-p53 [20]. It has been reported that inhibition of ASC expression could change the p53 expression level in vascular endothelial cells [16], and decreased ASC expression could inhibit phosphor-p53 levels in kerationcytes [17]. Here, we found that ASC deficient mice with HFD suppressed phosphor-p53 and p53 protein levels in SAT, inhibiting the expression of phosphor-AMPKα and AMPKα. It has been proposed that ablation of AMPKα leads to the development of obesity, and the phenotypes observed in *Asc^−/−^* mice were consistent with in vivo findings in *Ampkα^−/−^* mice [32]. The phenotypes observed in *Asc^−/−^* mice were consistent with previous in vivo and in vitro findings in *p53^−/−^* mice [33,34]. These data strongly evidenced that ASC deficiency promotes lipogenesis and inhibits lipolysis depending on p53/AMPK signaling.

Since the HFD feeding could not avoid the effects of inflammation, we also tested metabolic indicators in *Asc^−/−^* mice fed with normal-chow diet (ND). We found no significant metabolic differences between *Asc^−/−^* and LM mice, and the phosphor-p53, p53, phosphor-AMPKα, and AMPKα protein expression did not change either in the conditions of ND 12 weeks (Figure S2). We speculated that there was no excess energy stress for mice with a normal diet; the defect of ASC would not regulate the p53/AMPKα axis to affect lipid metabolism.

**Figure 5:**
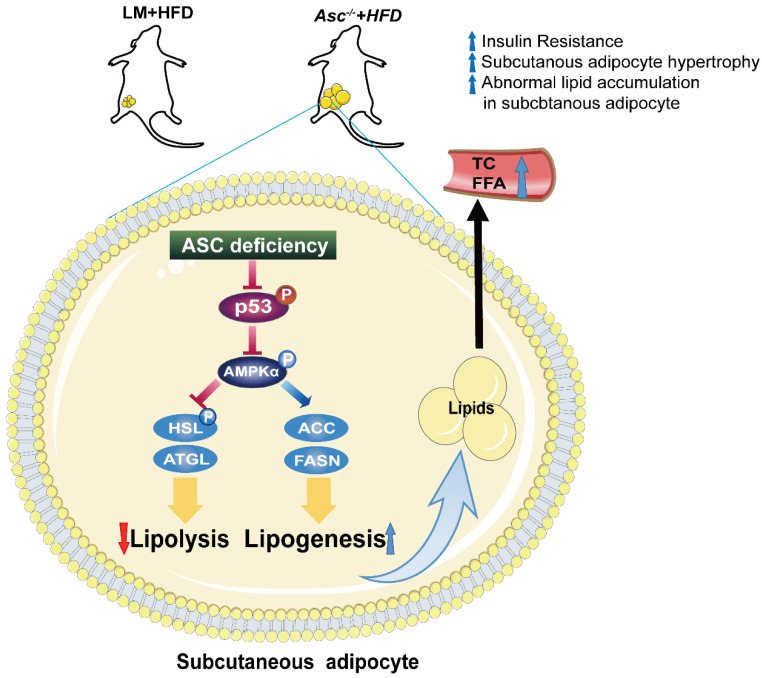
The proposed model for the mechanism by which ASC deficiency promotes lipid accumulation. A summary of our findings: ASC deficiency in SAT leads to decreased p53 expression, reducing AMPKα phosphorylation and lessening active AMPKα levels. ASC deficiency promotes lipogenesis, inhibits lipolysis, and causes increased subcutaneous adipocyte lipid accumulation, abnormal metabolism, and obesity.

Our findings provide insight into the effects of ASC via p53/AMPKα axis on lipid metabolism in SAT (Figure 5). When comparing our results to other studies, we must point out that our study focused more on cellular energy balance and lipid metabolism affected by ASC in adipocytes. There are also a few limitations in the present study that need to be considered. The first is that no *ASC^−/−^* patient was included in our research design due to the low incidence of *ASC* deficiency in humans and limited access to human samples. Still, a greater number of *ASC^−/−^* patients will be included as far as possible in our future experiments. Another limitation was that the outcomes on lipid metabolism when ASC is overexpressed need to be further studied.

## 4. Materials and Methods

### 4.1. Animals

Animal experiments were performed in accordance with the National Institutes of Health Guide for the Care and Use of Laboratory Animals with approval from the Ethics Committee of Chongqing Medical University. Wild-type C57/B6J (WT) mice were purchased from the Animal Center of Chongqing Medical University, and *Asc^−/−^* mice were kindly provided by Dr. Guocai Lu and Dr. Ying Zong (Department of Health Toxicology, Faculty of Naval Medicine, Second Military Medical University). Six-to-eight-week-old male transgenic mice (*Asc^−/−^*, n = 10 per group) and littermate mice (LM, n = 8 per group) were housed in colony cages under 12 h light and 12 h dark cycles at 23 ± 1 °C in the Laboratory Animal Center of Chongqing Medical University. All mice were on a standard diet for 2 weeks prior to the experiments, and then the mice were fed with a normal-chow diet (ND) (Beijing KEAO XIELI FEED, (2018)06073, Beijing, China)or high-fat diet (HFD),(Research Diet, D12492, 60% kcal fat) (diet composition: Fat: Lard and soybean Oil; Protein: Casein and Cystine; Carbohydrate: Lodex 10 and Sucrose; Fiber: Solka Floc; Mineral: S10026B; Vitamin Choline Bitartrate and V10001C; Dye: Blue FD&C #1) and water for 12 weeks, while the body weight of the mice was measured on the same day weekly, and the mice GTT/ITT were measured in the last week. Finally, the mice were sacrificed, the blood and SAT samples were collected for the follow-up experiments. All experiments were performed in the Laboratories Animal center of Chongqing Medical University.

### 4.2. Glucose Tolerance and Insulin Tolerance Tests

The mice chow was removed at 19:00 on the first day and the mice were fasted for 14–16 h. Then the body weight measured and fasting blood glucose (FBG) for a glucose tolerance test (GTT) at 9:00 the next morning. The mice were injected intraperitoneally with 2 mg/g glucose solution and blood glucose levels detected in tail blood by using a glucometer at 30, 60, 90, and 120 min after injection.

The mice chow was removed at 9:00 in the morning and the mice were fasted for 4–6 h, and then the body weight measured and FBG for insulin tolerance tested (ITT) at 13:00. The mice were injected intraperitoneally with 0.75 mIU/g insulin and blood glucose levels detected in tail blood by using a glucometer at 30, 60, 90, and 120 min after injection.

### 4.3. Biochemical Determination

Serum TC (Nanjing Jiancheng bioengineering Institute, A111-1-1, Nanjing, China), TG (Nanjing Jiancheng bioengineering Institute, A110-1-1, Nanjing, China) and NEFA (Nanjing Jiancheng bioengineering Institute, A042-2-1, Nanjing, China) levels were determined by using commercial kits and standard operation steps were according to the manufacturer’s protocol. Optical density (OD) was determined on a microplate reader. 

### 4.4. H&E Staining and Cell Size Quantitation

SAT was fixed in 4% paraformaldehyde, embedded in paraffin after dehydration with a series of ethanol solutions, and cut into slides with a thickness of 5-µm. Standard H&E staining was performed on 5-µm paraffin sections of SAT. Adipocyte size was calculated in the H&E-stained sections of 6–8 individual samples in each group by using Adipocount 1.1 accessed on 26 July 2020. Statistical analysis of adipocyte size: the Student *t*-test is used to compare the averages between 2 groups for significant differences; after the Frequency distribution test for adipocyte size frequency. The statistical significance threshold was set at 0.05.

### 4.5. Isolation of SVF

Stromal vascular fractions (SVF) from the adipose tissue were isolated as described previously [35]. Briefly, adipose tissue was harvested, cut into small pieces, and digested by collagenase (collagenase VIII) (Sigma, C2139, Saint Louis, MO, USA). After the digestion, the whole mixture was filtered in a 100 µm mesh filter, collected the whole SVF cells after centrifugation, and then removed red blood cells with an ammonium chloride lysis buffer.

### 4.6. Cell Culture

For adipocyte-progenitors differentiation, adipose tissue was dissected and digested as described above [35], plated in 3.5 cm dishes, adherent cells were grown to confluence. Differentiation is previously described. SVF cells were plated at low density (0.3 × 10^5^ cells/3.5 dish) and cultured in DMEM containing 10% (vol/vol) Fetal bovine serum (FBS) (Gibco, 04-002-1A, Grant Island, NY, USA), 1% penicillin/streptomycin (P/S) (Beyotime Biotechnology, C0222, Shanghai, China) at 37 °C with 5% CO_2_. Two days post-confluence (designated as day 0), cells were induced to differentiate with DMEM containing 10% (vol/vol) FBS, 5 μg/mL insulin (Roche, San Francisco, CA, USA), 1 μM dexamethasone (Sigma, Saint Louis, MO, USA), and 0.5 mM 3-isobutyl-1-methyl-xanthine (Sigma, Saint Louis, MO, USA) until day 2. Cells then were fed with DMEM supplemented with 10% (vol/vol) FBS and 5 μg/mL insulin for 2 d, after which they were fed every other day with DMEM containing 10% (vol/vol) FBS. For activator treatment, cells were incubated with 500 µM AICAR (Selleck, S1802, Houston, TX, USA) or 340 µM Kevetrin (Selleck, S5811, Houston, TX, USA) for 5–24 h for protein and Oil red O staining.

### 4.7. siRNA and Transfection

Pre-designed ASC and control scrambled siRNA were purchased from Gene Pharma (Shang Hai, China): AAGATGCGGAAGCTCTTCAGT (nucleotides472-592) for ASC [36]. All transfection was performed using the Lipofectamine™ RNAiMAX™ system (Thermo Fisher Scientific, 13778150, Waltham, MA, USA), following the manufacturer’s protocol. Transient knockdown of ASC was performed in primary SVF cells. For ASC siRNA treatment, 25 pmol siRNA was added to the medium during the differentiation from day 4.

### 4.8. Oil Red O Staining

SVF cells were stained with Oil red O (ORO) (Sigma, O0625-100G, Saint Louis, MO, USA) as described previously [37]. They were washed three times with phosphate-buffered saline (PBS) and fixed for 60 min in buffered 4% formalin. ORO was diluted with water (3:2), filtered through a 0.45-µm filter, and incubated with the cells for 2 h at room temperature. Then, the cells were washed with water, visualized, and photographed with light microscopy.

### 4.9. Flow Cytometry Analysis

Adipose tissues were minced in PBS containing 0.075% collagenase (Sigma-Aldrich, C2139, Saint Louis, MO, USA). After being incubated at 37 °C for 30 min and filtrated with a 100-mesh filter, cell suspensions were centrifuged at 1500 rpm for 5 min to remove adipocytes. Isolated stromal vascular fraction (SVF) pellet was collected from the bottom. The SVF pellet was resuspended in PBS containing 3% BSA, then red blood cell lysis buffer was added and incubated for 3 min. After washing in 3% BSA, the bottom cells were incubated as described previously [38].

### 4.10. Quantitative Real-Time PCR

Total RNA was extracted from SAT or SVF cells using Trizol reagent (Thermo Fisher Scientific, 15596026, Waltham, MA, USA). From each sample, 1.5 µg of total RNA was reverse-transcribed into cDNA using the Revert Aid First Strand cDNA Synthesis Kit (Thermo Fisher Scientific, 00698284, Waltham, MA, USA) with T100 (Bio-Rad, Hercules, CA, USA) according to the manufacturer’s instructions. The mRNA levels of the investigated genes were measured using SYBR Green Master Mix (Thermo Fisher Scientific, 00736756, Waltham, MA, USA), normalized to 18 s, and analyzed by 2^−ΔΔct^ with Quantstudio3/5 (Thermo Scientific, Waltham, MA, USA) real-time PCR instrument. The primer sequences used are shown in Table 1.

### 4.11. Western Blot Analysis

Cells were homogenized in cell lysis buffer (10% sodium dodecyl sulfate (SDS) and 50 mM Tris.HCl (pH 6.8) on ice for 10 min. The tissue samples were sonicated (70 Hz, 90 s) in cell lysis buffer, followed by an additional incubation on ice for 20 min. Then, the lysates were centrifuged at 4 °C for 15 min at 12,000 rpm. The protein denaturation was accomplished by incubation at 100 °C. Equal amounts (30 µg) of protein samples were separated by 10% SDS-PAGE gels, separated by electrophoresis, and subsequently transferred onto PVDF membranes. After being blocked with 5% skim milk, the membranes were incubated with the primary antibodies, which were diluted with 5% bovine serum albumin (BSA) (Sangon Biotech, A602440-0050, Shanghai, China) at 4 °C overnight, and then appropriate secondary HRP-conjugated antibodies at room temperature 1 h. Antibodies are shown in Table 2.

### 4.12. Statistical Analysis

All data were presented as means ± SEM and analyzed in Graph Pad Prism 7 (GraphPad Software, San Diego, CA, USA). Comparisons between two groups were analyzed using the unpaired two-tailed Student’s *t* test. Pearson correlation coefficient analysis was used to evaluate the correlation between the relative expression of genes. *p* < 0.05 indicates statistical significance. All experiments were repeated at least three times.

## 5. Conclusions

In conclusion, the ablation of ASC leads to metabolic disorders and obesity when animals are fed with HFD due to an enhanced lipid accumulation in subcutaneous adipocytes. Our study confirms that ASC deficiency can promote lipogenesis and inhibit lipolysis via p53/AMPKα axis in vivo and in vitro. Therefore, our findings help understand ASC deficiency-related obesity and add new knowledge about the role of ASC protein in the control and regulation of lipid metabolism.

## Figures and Tables

**Table 1:** Primers for qRT-PCR.

Primers	Sequence (5′-3′)	
*Asc*	Forward	CTTGTCAGGGGATGAACTCAAAA
	Reverse	GCCATACGACTCCAGATAGTAGC
*Fabp4*	Forward	GCGTAAATGGGGATTTGGTC
	Reverse	CTCCTGTCGTCTGCGGTGATT
*Fasn*	Forward	AGGTGGTGATAGCCGGTATGT
	Reverse	TGGGTAATCCATAGAGCCCAG
*18s*	Forward	CGCCGCTAGAGGTGAAATTCT
	Reverse	CATTCTTGGCAAATGCTTTCG
*Acc*	Forward	CACCAGTTTTGCATTGAGAAC
	Reverse	TACGCTGTTGAGTTCATAGGC
*Srebp1c*	Forward	GGAGCCATGGATTGCACATT
	Reverse	CAGGAAGGCTTCCAGAGAGG
*Scd1*	Forward	CTCTACACCTGCCTCTTCGG
	Reverse	GCCGTGCCTTGTAAGTTCTG
*Cd36*	Forward	TGGTCAAGCCAGCTAGAAA
	Reverse	TCCCAAGTAAGGCCATCTC
*Lxrα*	Forward	GCCTACAGAACTTCGTCCACA
	Reverse	AAGAATCCCTTGCAGCCCTC
*Mcd*	Forward	GGGGCTGTGATGTGGCGTAT
	Reverse	GGGCTACCAGGCTGAGGAT
*Dgat1*	Forward	GTTTCCGTCCAGGGTGGTAGT
	Reverse	TGGCACCTCAGATCCCAGTAG

**Table 2:** Antibodies used in this paper.

Antibody	Company	Cat Number	Dilution
α-Tubulin	Cell Signaling Technology	2125S	1:1000
ASC	Cell Signaling Technology	67824	1:2000
FASN	Proteintech	66591-1-1g	1:1000
ACC	Proteintech	21923-1-AP	1:1000
FABP4	Affinity	DF6035	1:1000
p-HSL	Novus	NBP3-05459	1:1000
HSL	Cell Signaling Technology	4107S	1:1000
ATGL	Cell Signaling Technology	2439S	1:1000
p53	Santa Cruz Technology	SC-126	1:1000
p-p53	Abmart	T40061	1:1000
AMPKα	Santa Cruz Technology	SC-74464	1:1000
p-AMPKα	Cell Signaling Technology	2535S	1:1000
p-p65	Cell Signaling Technology	3033S	1:1000
p65	Cell Signaling Technology	8242S	1:1000
IL-1β	Santa Cruz Technology	SC-12742	1:1000

## Data Availability

Not applicable.

## Supplementary Materials

The following supporting information can be downloaded at: https://www.mdpi.com/article/10.3390/ijms231710042/s1.
