# Supplementary material for: ASC Regulates Subcutaneous Adipose Tissue Lipogenesis and Lipolysis via p53/AMPKα Axis
Source: Int J Mol Sci. 2022 Sep 2;23(17):10042. doi: 10.3390/ijms231710042 (PMC9456541; doi:10.3390/ijms231710042)
Supplement: Supplementary file 1 [file ijms-23-10042-s001.zip › ijms-1862000-supplementary.pdf]

Supplementary Materials

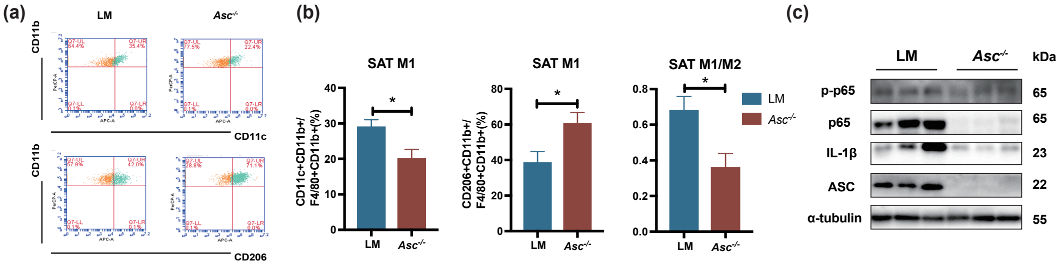

Supplementary Figure S1. Inflammation level and ratio of M1/M2 ATMs were decreased in SAT of *Asc*$^{-/-}$ mice with HFD.

(a, b). FCM analysis of SAT of LM and *Asc*$^{-/-}$ mice which fed with HFD. (12 weeks). (n=5/group) **(a).** Representative plots of FCM analysis: proportion of M1 or M2 macrophages to ATMs. **(b).** Statistics of FCM analysis. Data are percentages of M1ATMs, percentages of M2 ATMs, and M1/M2 ratios. **(c).** WB of phosphorylated p65 (p-p65), p65 and IL-1β proteins in SAT of mice. All the mice were male and fed with HFD for 12 weeks, if not indicated otherwise. n=6-10/group. *P<0.05, **P<0.01, and***P<0.001.

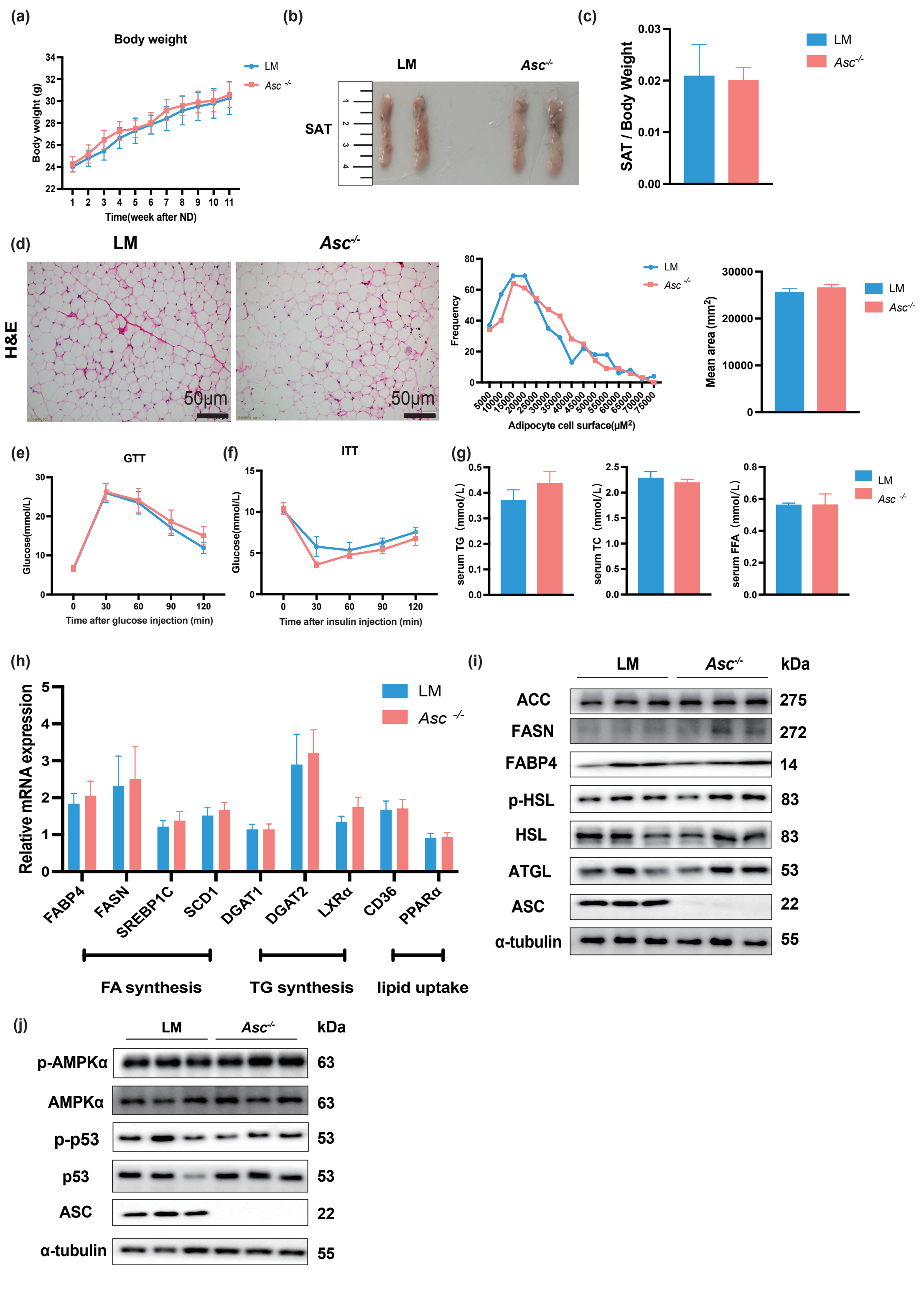

Supplementary Figure S2. In normal diet, ASC deficiency did not affect lipogenesis and lipolysis in SAT.

(a). Body weight of LM(n=6/group) and *Asc* knockout (*Asc*$^{-/-}$) mice(n=10/group), fed with ND (12 weeks). **(b).** Comparison of SAT with each group. **(c).** Fat index (ratio of SAT weight to whole body

weight) of indicate mice. **(d).**H&E and quantification of SAT cell size. **(e, f).** GTT and ITT analysis. **(g).** Plasma concentrations of TG, TC and NEFA at baseline. **(h).** mRNA expression and protein level of lipogenesis genes in SAT. **(i).** Western blot analysis of lipogenesis and lipolysis protein in SAT. **(j).** Western blot analysis of phosphor-p53, p53, phosphor-AMPKα and AMPKα in SAT. All the mice were male and fed with ND for 12weeks, if not indicated otherwise. n=6-10/group. *P<0.05, **P<0.01, and***P<0.001.
